# COVID-19 vaccine coverage and factors associated with vaccine uptake among individuals with a recent experience of homelessness: a population-based analysis from Ontario, Canada.?

**DOI:** 10.23889/ijpds.v7i3.1807

**Published:** 2022-08-25

**Authors:** Shariff Salimah, Lucie Richard, Stephen Hwang, Jeffrey Kwong, Cheryl Forchuk, Naheed Dosani, Richard Booth

**Affiliations:** 1 ICES; 2 MAP Centre for Urban Health Solutions; 3 Western University Canada; 4 University of Toronto

## Objectives

To describe COVID-19 vaccine coverage (i.e., the estimated percentage of people who have received a vaccine) and determinants of vaccine receipt among individuals with a recent experience of homelessness in Ontario, Canada.

## Approach

We conducted a retrospective, population-based cohort study of 23,247 individuals (≥18 years) with a recent experience of homelessness as recorded in routinely collected healthcare databases. Participants were followed from December 14, 2020 to September 30, 2021 for the receipt of a COVID-19 vaccine. Using modified Poisson regression, we identified sociodemographic, healthcare usage, and clinical factors associated with the receipt of one or more doses of a COVID-19 vaccine.

## Results

By September 30, 14,271 (61.4%) of participants with a recent experience of homelessness had received a first dose of a COVID-19 vaccine and 11,082 (47.7%) had received two doses. Over the same period, 86.6% and 81.6% of the total adult population of Ontario had received a first dose and second dose, respectively. In multivariable analysis, factors associated with increased COVID-19 uptake included ≥1 visit to a general practitioner (adjusted Risk Ratio [aRR]:1.37[95% CI 1.31-1.42]), older age (vs. 18-29 years: 50-59 years, aRR:1.18[1.14-1.22]; 60+ years, aRR:1.27[1.22-1.31]), receipt of an influenza vaccine (aRR:1.25[1.23-1.28]), receipt of ≥1 SARS-CoV-2 test (aRR:1.23[1.20-1.26]) and the presence of chronic health conditions (vs. 0 conditions: 1 condition, aRR:1.05[1.03, 1.08]; 2+ conditions, aRR:1.11[1.08-1.14]). In contrast, living in a smaller metropolitan region (aRR:0.92[0.90-0.94]) or rural location (aRR:0.93[0.90-0.97]) compared to a large metropolitan region was associated with lower uptake.

## Conclusion

As of September 30, 2021, COVID-19 vaccine coverage among individuals with a recent experience of homelessness in Ontario was substantially lower than the general adult population of Ontario for a first and second dose. Findings underscore the importance of leveraging organizations that are accessed and trusted by people who experience homelessness for targeted vaccine delivery.

